# Maternal residential proximity to unconventional gas development and perinatal outcomes among a diverse urban population in Texas

**DOI:** 10.1371/journal.pone.0180966

**Published:** 2017-07-21

**Authors:** Kristina W. Whitworth, Amanda K. Marshall, Elaine Symanski

**Affiliations:** 1 Department of Epidemiology, Human Genetics and Environmental Sciences, UTHealth School of Public Health in San Antonio, San Antonio, Texas, United States of America; 2 Southwest Center for Occupational and Environmental Health, UTHealth School of Public Health, Houston, Texas, United States of America; 3 Department of Epidemiology, Human Genetics and Environmental Sciences, UTHealth School of Public Health, Houston, Texas, United States of America; Stony Brook University, Graduate Program in Public Health, UNITED STATES

## Abstract

**Objective:**

To assess associations between unconventional natural gas development (UGD) and perinatal outcomes.

**Methods:**

We conducted a retrospective birth cohort study among 158,894 women with a birth or fetal death from November 30, 2010-November 29, 2012 in the Barnett Shale, in North Texas. We constructed three UGD-activity metrics by calculating the inverse distance-weighted sum of active wells within three separate geographic buffers surrounding the maternal residence: ≤½, 2, or 10-miles. We excluded women if the nearest well to her residence was >20 miles. Metrics were categorized by tertiles among women with ≥1 well within the respective buffer; women with zero wells ≤10 miles (the largest buffer) served as a common referent group. We used logistic or linear regression with generalized estimating equations to assess associations between UGD-activity and preterm birth, small-for-gestational age (SGA), fetal death, or birthweight. Adjusted models of fetal death and birthweight included: maternal age, race/ethnicity, education, pre-pregnancy body mass index, parity, smoking, adequacy of prenatal care, previous poor pregnancy outcome, and infant sex. Preterm birth models included all of the above except parity; SGA models included all of the above except previous poor pregnancy outcome.

**Results:**

We found increased adjusted odds of preterm birth associated with UGD-activity in the highest tertiles of the ½- (odds ratio (OR) = 1.14; 95% confidence interval 1.03, 1.25), 2- (1.14; 1.07, 1.22), and 10-mile (1.15; 1.08, 1.22) metrics. Increased adjusted odds of fetal death were found in the second tertile of the 2-mile metric (1.56; 1.16, 2.11) and the highest tertile of the 10-mile metric (1.34; 1.04–1.72). We found little indication of an association with SGA or term birthweight.

**Conclusions:**

Our results are suggestive of an association between maternal residential proximity to UGD-activity and preterm birth and fetal death. Quantifying chemical and non-chemical stressors among residents near UGD should be prioritized.

## Introduction

Advancements in horizontal drilling and hydraulic fracturing have increased access to previously untapped natural gas reserves in shale formations. Unconventional natural gas development (UGD) is associated with several potential environmental hazards. The hydraulic fracturing process involves injecting a pressurized mixture of sand, water, and proprietary fracking fluid into wellbores, fracturing the rock and unlocking trapped hydrocarbons [1]. Fracking fluid may contain compounds that are known or possible human carcinogens, regulated under the Safe Drinking Water Act, or classified as hazardous air pollutants [2]. Ground and surface water contamination can occur from migration of fluids through failed well casings, leakage from open pit storage, and improper disposal or treatment of wastewater [3–7]. Further, many compounds found in fracking fluid and wastewater have been indicated for their reproductive or developmental toxicity [8, 9]. Multiple air pollutants including volatile organic compounds (VOCs) (e.g., toluene, benzene), polycyclic aromatic hydrocarbons (e.g., naphthalene, benzo(a)pyrene), nitrogen oxides, ozone, and particulate matter have also been detected near unconventional drilling sites [6, 10–14]. In addition to potential chemical exposures, individuals living in communities near UGD may experience noise and light pollution, noxious odors, and increased psychosocial stressors [15, 16]. Non-chemical stressors can contribute to allostatic load, reducing overall health and wellbeing [17], and potentially increase susceptibility to chemical stressors [18].

Results of the few epidemiologic studies of the association between maternal residential proximity to UGD and perinatal outcomes are equivocal [19–21]. Although exposure timing is a critical consideration in such studies, only one of these previous studies limited analyses to UGD-activity occurring specifically during the gestational period [21]. The remaining two studies captured all UGD-activity during the year of the child’s birth [19, 20]. Additionally, previous studies have been conducted among mostly white, mostly rural populations. Our study includes women living in the Barnett Shale, one of the oldest and most developed shale plays in the United States. UGD-activity in the Barnett Shale is concentrated in and around the Dallas-Fort Worth metroplex, the fourth largest metropolitan statistical area in the nation and home to a heterogeneous population [22]. Our goal was to assess the association between maternal residential proximity to UGD-activity and perinatal outcomes, considering timing of UGD-activity relative to pregnancy. Due to the dearth of data informing: (a) the most relevant distances within which to capture impacts of chemical and non-chemical stressors related to UGD and (b) implications of such decisions on health effect estimates, we also examined the characterization of proximity to UGD-activity according to several distance criteria.

## Materials and methods

This retrospective birth cohort study included women with a singleton birth or fetal death from November 30, 2010-November 29, 2012 in the 24-county Barnett Shale area (Archer, Bosque, Clay, Comanche, Cooke, Coryell, Dallas, Denton, Eastland, Ellis, Erath, Hill, Hood, Jack, Johnson, Montague, Palo Pinto, Parker, Shackelford, Somervell, Stephens, Tarrant, Wise, and Young counties), in North Texas. Birth and fetal death records were obtained from the Texas Department of State Health Services (TXDSHS) for 166,966 births and 866 fetal deaths. This study was approved by the Committee for Protection of Human Subjects at The University of Texas Health Science Center at Houston and the TXDSHS IRB.

We corrected implausible birthweight for gestational age combinations for live births, according to methods previously described [23–25]. For live births, we estimated the conception date by subtracting gestational age from the child’s birth date. For fetal deaths, we estimated the conception date by subtracting the last menstrual period (LMP)-based estimate of gestational age from the date of death; for records missing the LMP-based estimate (n = 223), we used the clinical estimate. We defined small-for-gestational age (SGA; yes/no) as birthweight for gestational age ≤10^th^ percentile of the sex-specific weight for age distribution in our study sample. Preterm birth (yes/no) was defined as a live birth delivered before 37 completed weeks gestation. We identified fetal deaths from death records (yes/no). We obtained birthweight (g) from birth records and treated it as a continuous outcome.

Street-level geocoded location of maternal residence at birth was provided by TXDSHS [26] for the majority of records and we manually geocoded physical addresses for the remaining records using ArcMap (v. 10.2.1; ESRI, Redlands CA). We obtained the following covariates from birth/fetal death records: maternal age (≤20, 21–25, 26–30, 31–35, >35 years), education (<high school, high school graduate, some college, college graduate), parity (0, ≥1), smoking during pregnancy (yes/no), race/ethnicity (non-Hispanic white, non-Hispanic black, Hispanic, other), pre-pregnancy body mass index (BMI: ≤18.5 kg/m^2^, 18.5–24.9 kg/m^2^, 25.0–29.9 kg/m^2^, 30.0–34.9 kg/m^2^, ≥35.0 kg/m^2^), infant sex, and previous poor pregnancy outcome (i.e., previous perinatal death, intrauterine growth restriction, pregnancy termination, preterm birth, or SGA; yes/no). We also constructed the Adequacy of Prenatal Care Utilization Index (inadequate, intermediate, adequate, adequate plus, unknown) which captures timing of first prenatal visit and frequency of visits [27]. The ‘unknown’ category includes women for whom date of first visit or number of visits was missing, but for whom records indicated prenatal care was received. The ‘adequate plus’ category indicates receipt of more than the recommended number of visits (i.e., one visit/month for weeks up to 28, two visits/month for weeks 29 to 36, and weekly visits from 37 weeks on, as outlined in Kotelchuck [27]), presumably due to high-risk pregnancies. Finally, we calculated the exact geodesic line-distance from the residence to the nearest major roadway as a proxy for traffic-related air pollution (<300m, ≥300m) [28].

We obtained UGD data from DrillingInfo (www.drillinginfo.com), a commercial site which maintains a national database of oil and gas well locations and characteristics (updated twice monthly) [29] on May 12, 2015. We identified unconventional (i.e., horizontal/directionally drilled) gas wells in the Barnett Shale with either spud (i.e., earliest known date ground was broken in the process of well development), completion (i.e., date when installation of the well casing, pumping mechanism, and hydraulic fracturing were completed) [30] or production dates between January 1, 2010–November 29, 2012. Wells can be completed more than once, often to stimulate production, and multiple completion dates may be reported [30]. In this case, we retained the most recent date. We captured active wells beginning January 1, 2010 to characterize UGD-activity for the entire pregnancy for all births in the cohort. We did not include wells that had a permit date but no record of other activity. In total, we identified 14,351 unique active UGD wells.

We constructed three separate exposure metrics by generating geographic buffers around each residence, at distances of ½, 2, and 10 miles. One-half mile was chosen on the basis of a previous risk assessment [31], 10 miles was chosen to be consistent with prior studies [19, 20], and 2 miles was chosen as an intermediate. For each woman’s residence, we calculated the inverse distance-weighted (IDW) sum of active UGD wells within each buffer according to:

$$IDW_{a}=\overset{n}{\underset{i=1}{\sum }}\frac{1}{d_{i}^{2}}$$

where ‘a’ indicates buffer distance, ‘i’ is a given well in the specified buffer, ‘d’ is the exact geodesic line distance between that well and the residence, and ‘n’ is the total number of wells in the specified buffer. Because women living very far from UGD wells likely differ from women living near UGD activity, women for whom the nearest well was >20 miles from the residence were excluded. We then categorized each of the three metrics by tertiles among women with ≥1 well within the respective buffer. To enable comparison of effect estimates across the three metrics, we chose a common referent group for all analyses: women with zero wells ≤10 miles of her residence (given exclusion of women for whom the nearest well was >20 miles, this group effectively represents women for whom the nearest well is ≥10 miles but >20 miles away).

We used logistic regression to examine the relation between each UGD-activity metric and preterm birth, SGA, and fetal death and linear regression to examine the relation with birthweight. Given potential correlation among women within census-tracts, we applied generalized estimating equations to all models, assuming an exchangeable correlation structure and treating census tract as a random effect. We included maternal age, pre-pregnancy BMI, and maternal race/ethnicity in all models *a priori*. We identified additional covariates separately for each outcome. Covariates which were statistically significantly (p<0.05) associated with the respective outcome were included in the final adjusted model. In this way, a common set of variables were included in all adjusted models for each outcome, irrespective of the UGD-metric, facilitating comparison across metrics. In addition to the *a priori* variables, education, parity, smoking status, infant sex, previous poor pregnancy outcome, and the Adequacy of Prenatal Care Utilization Index were included in models of birthweight and fetal death. The preterm birth models did not include parity and SGA models did not include previous poor pregnancy outcome.

Given the association of maternal residential distance to the nearest major roadway with pregnancy outcomes in previous studies [32], we conducted a sensitivity analysis including this variable in the adjusted models. In a second sensitivity analysis, we controlled for season of conception, categorized as October-March or April-September, given the weather patterns in Texas. All analyses were performed using SAS version 9.4 (SAS Institute Inc., Cary, NC) or ArcGIS version 10.2 (ESRI, Redlands, CA).

## Results

**Fig 1:**
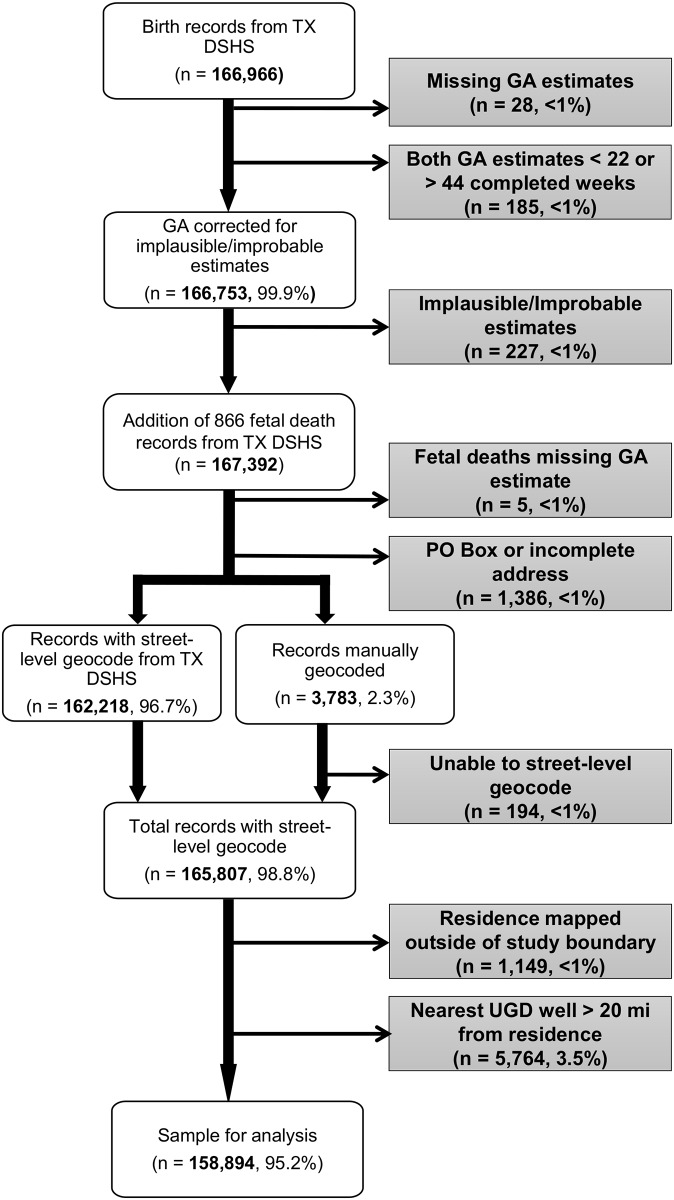
Flow chart outlining study process and exclusions among women living in the 24-county Barnett Shale area with a birth or fetal death between Nov. 30, 2010 and Nov. 29, 2012. Note: GA = Gestational Age; TX DSHS = Texas Department of State Health Services.

Fig 1 outlines the study process and exclusions. Briefly, through the process of cleaning gestational age [23–25], we excluded 28 (<1%) births missing both LMP- and clinical-based estimates of gestational age as well as 185 (<1%) births with estimated gestational age <22 or >44 completed weeks (Fig 1). A total of 227 births were excluded due to implausible/improbable gestational age estimates. We excluded five fetal deaths with no estimate of gestational age. Among records with a street-level geocode, 1,149 (<1%) were located outside the study area and were also excluded. An additional 5,764 (3.5%) subjects were excluded because the nearest UGD well was >20 miles from the residence. The final sample was 158,894: 158,104 live births and 790 fetal deaths.

Women in this study were mostly young (31.5% were >30 years) with at least a high school education (79.1%) and did not smoke during pregnancy (95.6%) (Table 1). Hispanics (39.7%) comprised the largest racial/ethnic group, followed by non-Hispanics whites (37.4%) and Blacks (16.1%). More than one-third (36.2%) of women had less than adequate prenatal care and 18.1% received adequate plus care, suggesting higher risk pregnancies.

**Table 1:** Characteristics of 158,894 women with a singleton birth or fetal death in the 24-county Barnett Shale area, Texas, Nov. 30, 2010-Nov. 29, 2012.

Characteristic	n (%)
**Maternal age (years)**	
≤20	23,890 (15.0)
21–25	39,744 (25.0)
26–30	45,300 (28.5)
31–35	33,797 (21.3)
>35	16,163 (10.2)
**Race/ethnicity**	
Non-Hispanic White	59,400 (37.4)
Non-Hispanic Black	25,555 (16.1)
Hispanic	63,172 (39.7)
Other	10,767 (6.8)
**Pre-Pregnancy BMI (kg/m**^**2**^**)**	
< 18.5	5,973 (3.8)
18.5–24.9	82,436 (52.3)
25.0–29.9	36,192 (22.9)
30.0–34.9	19,164 (12.1)
≥35.0	13,984 (8.9)
*Missing*	*1*, *145* (*0*.*7*)
**Maternal Education**	
< High School	33,221 (20.9)
High School Grad	48,521 (30.6)
Some College	38,995 (24.6)
College Degree	38,042 (23.9)
*Missing*	*115 (<0*.*1)*
**Parity**	
0	63,355 (39.9)
≥1	95,503 (60.1)
*Missing*	*36 (<0*.*1)*
**Smoked During Pregnancy**	
No	150,979 (95.6)
Yes	6,919 (4.4)
*Missing*	*996 (0*.*6)*
**Adequacy of Prenatal Care Utilization**	
Inadequate	34,111 (21.5)
Intermediate	23,434 (14.7)
Adequate	65,463 (41.2)
Adequate Plus	28,708 (18.1)
Unknown	7,178 (4.5)
**Previous Poor Pregnancy Outcome**	
No	156,207 (98.3)
Yes	2,687 (1.7)
**Infant Gender**	
Male	81,388 (51.2)
Female	77,504 (48.8)
*Missing*	*2 (<0*.*1)*
**Small-for-Gestational Age**^1^	
No	137,466 (87.0)
Yes	20,638 (13.0)
**Preterm Birth**^1^	
No	145,017 (91.7)
Yes	13,087 (8.3)
**Fetal Death**	
No	158,104 (99.5)
Yes	790 (0.5)
**Birthweight**^2^ **(grams), Median ± IQR**	3364 ± 292

kg/m2, kilograms per meter squared; IQR, interquartile range.

^1^n = 158,104 because fetal deaths were excluded

^2^n = 145,017 because fetal deaths and preterm births were excluded

The proportion of women with ≥1 active UGD well near her residence during pregnancy varied by distance within which UGD was captured: 15.9% at ½ mile, 45.1% at 2 miles, and 75.8% at 10 miles. The median number of proximal wells during pregnancy increased with buffer size: three ≤½ mile, 28 ≤2 miles and 413 ≤10 miles. This divergence was more apparent at the extremes of the distribution: maximum wells ≤10 miles of the residence during pregnancy was >2,000, versus 32 wells ≤½ mile. We observed similar patterns in the distribution of IDW metrics (Table 2).

**Table 2:** Distribution^1^ of the number and IDW sum of active UGD wells near women's residences during pregnancy, among 158,894 women with a singleton birth or fetal death in the 24-county Barnett Shale area, Texas, Nov. 30, 2010—Nov. 29, 2012, by buffer size.

Buffer	25%	50%	75%	95%	Max
**½ Mile**					
Count	2	3	6	12	32
IDW Sum	13.4	31.4	70.1	208.2	13447.3
**2 Miles**					
Count	12	28	47	92	168
IDW Sum	7.9	24.0	51.2	151.0	13145.6
**10 Miles**					
Count	70	413	1,048	1,637	2,374
IDW Sum	1.0	19.2	63.6	163.3	13480.1

IDW: inverse distance weighted; UGD: unconventional gas development; Max: maximum.

^1^Calculated among women with ≥1 well within the specified buffer

Crude associations between UGD-activity and preterm birth were largely null for each UGD-metric. After adjustment, we found increased odds of preterm birth associated with UGD-activity in the highest tertiles of the ½- (odds ratio (OR) 1.14; 95% confidence interval (CI) 1.03, 1.25), 2- (OR 1.14; 95% CI 1.07, 1.22), and 10- (OR 1.15; 95% CI 1.08, 1.22) mile metrics (Table 3). The highest odds of preterm birth were found among women classified in the second tertile of the ½- mile metric compared to women with zero wells ≤10 miles of her residence (OR 1.21, 95% CI 1.09, 1.33).

**Table 3:** Crude and adjusted^1^ associations between UGD-activity and adverse birth outcomes, among 156,697 women with a birth or fetal death in the 24-county Barnett Shale area, Texas, Nov. 30, 2010—Nov. 29, 2012.

IDW Sum of UGD Well Activity	No.	Cases	Median (IQR) Wells	Crude OR (95% CI)	Adjusted OR (95% CI)
**½ *Mile Buffer***					
**Preterm Birth**^2^					
0 Wells ≤10 mi	37,885	3,183		Reference	
1^st^ Tertile	8,161	682	1 (1–2)	0.99 (0.91, 1.08)	1.18 (1.08, 1.29)
2^nd^ Tertile	8,412	717	4 (3–5)	1.02 (0.93, 1.11)	1.21 (1.09, 1.33)
3^rd^ Tertile	8,144	654	7 (5–10)	0.95 (0.87, 1.04)	1.14 (1.03, 1.25)
**SGA**^3^					
0 Wells ≤10 mi	37,882	5,361		Reference	
1^st^ Tertile	8,161	964	1 (1–2)	0.81 (0.75, 0.87)	0.95 (0.89, 1.00)
2^nd^ Tertile	8,409	1,063	4 (3–5)	0.88 (0.82, 0.94)	1.01 (0.96, 1.06)
3^rd^ Tertile	8,142	1,013	7 (5–10)	0.86 (0.80, 0.93)	1.01 (0.96, 1.06)
**Fetal Death**^4^					
0 Wells ≤10 mi	38,029	147		Reference	
1^st^ Tertile	8,188	27	1 (1–2)	0.85 (0.57, 1.29)	1.07 (0.69, 1.65)
2^nd^ Tertile	8,438	29	4 (3–5)	0.89 (0.60, 1.32)	1.10 (0.72, 1.71)
3^rd^ Tertile	8,174	32	7 (5–10)	1.01 (0.69, 1.49)	1.27 (0.82, 1.97)
***2 Mile Buffer***					
**Preterm Birth**^2^					
0 Wells ≤10 mi	37,885	3,183		Reference	
1^st^ Tertile	23,231	1,856	7 (3–13)	0.95 (0.89, 1.01)	1.11 (1.04, 1.19)
2^nd^ Tertile	23,758	2,006	32 (23–42)	1.01 (0.95, 1.07)	1.16 (1.09, 1.24)
3^rd^ Tertile	23,227	1,921	54 (39–75)	0.98 (0.93, 1.04)	1.14 (1.07, 1.22)
**SGA**^3^					
0 Wells ≤10 mi	37,882	5,361		Reference	
1^st^ Tertile	23,227	2,785	7 (3–13)	0.83 (0.79, 0.87)	0.95 (0.90, 1.00)
2^nd^ Tertile	23,757	2,984	32 (23–42)	0.87 (0.83, 0.91)	0.96 (0.91, 1.01)
3^rd^ Tertile	23,223	2,847	54 (39–75)	0.85 (0.81, 0.89)	0.95 (0.90, 1.00)
**Fetal Death**^4^					
0 Wells ≤10 mi	38,029	147		Reference	
1^st^ Tertile	23,301	74	7 (3–13)	0.82 (0.62, 1.09)	1.14 (0.83, 1.56)
2^nd^ Tertile	23,860	103	32 (23–42)	1.12 (0.87, 1.44)	1.56 (1.16, 2.11)
3^rd^ Tertile	23,300	77	54 (39–75)	0.85 (0.65, 1.13)	1.16 (0.86, 1.58)
***10 Mile Buffer***					
**Preterm Birth**^2^					
0 Wells ≤10 mi	37,885	3,183		Reference	
1^st^ Tertile	39,169	3,140	10 (1–67)	0.95 (0.90, 1.00)	1.02 (0.96, 1.08)
2^nd^ Tertile	40,143	3,296	418 (267–748)	0.98 (0.93, 1.03)	1.13 (1.06, 1.20)
3^rd^ Tertile	38,922	3,253	1190 (923–1489)	0.99 (0.95, 1.05)	1.15 (1.08, 1.22)
**SGA**^3^					
0 Wells ≤10 mi	37,882	5,361		Reference	
1^st^ Tertile	39,168	5,233	10 (1–67)	0.94 (0.90, 0.98)	1.03 (0.98, 1.08)
2^nd^ Tertile	40,139	4,924	418 (267–748)	0.85 (0.81, 0.88)	0.95 (0.91, 1.00)
3^rd^ Tertile	38,917	4,877	1190 (923–1489)	0.87 (0.83, 0.91)	0.96 (0.92, 1.01)
**Fetal Death**^4^					
0 Wells ≤10 mi	38,029	147		Reference	
1^st^ Tertile	39,325	157	10 (1–67)	1.03 (0.83, 1.29)	1.26 (0.99, 1.60)
2^nd^ Tertile	40,277	138	418 (267–747)	0.89 (0.70, 1.12)	1.22 (0.95, 1.57)
3^rd^ Tertile	39,066	149	1190 (923–1490)	0.99 (0.79, 1.24)	1.34 (1.04, 1.72)

UGD: unconventional gas development; IDW: inverse distance weighted; IQR: interquartile range; OR: odds ratio; CI: confidence interval; mi: miles.

^1^All models adjusted for maternal age at delivery, pre-pregnancy BMI, race/ethnicity, education, smoking, adequacy of prenatal care utilization, and infant sex.

^2^Preterm Birth models additionally adjusted for previous poor pregnancy outcome; n = 156,119 because fetal deaths are excluded.

^3^SGA models additionally adjusted for parity; n = 156,106 because fetal deaths excluded.

^4^Fetal death models additionally adjusted for parity and previous poor pregnancy outcome.

We found indication of weak inverse associations between UGD-activity and SGA within each distance in the crude models (Table 3). Little evidence of an association was observed in adjusted models, though, as results from adjusted models were attenuated (Table 3).

Little evidence of association between UGD-activity and fetal death was observed in crude models (Table 3). Though the estimate was imprecise, we found increased adjusted odds of fetal death among women classified in the highest tertile of UGD-activity for the ½-mile metric (OR 1.27, 95% CI 0.82, 1.97) and in the 2^nd^ tertile of the 2-mile metric (OR 1.56, 95% CI 1.16, 1.58). We observed the strongest association between UGD-activity and fetal death using the 10-mile metric. We observed increased adjusted odds of fetal death among women in each tertile of the 10-mile UGD-activity metric: 1^st^ tertile OR 1.26 (95% CI 0.99, 1.60), 2^nd^ tertile OR 1.22 (95% CI 0.95, 1.57), 3^rd^ tertile OR 1.34 (95% CI 1.04, 1.72).

Crude models between UGD-activity and birthweight revealed positive associations (Table 4). However, after adjusting for confounders, we found negative associations that were only moderate in strength. For example, compared to women with zero wells ≤10-miles of her home, we found an 8.20 g decrease (95% CI -18.36, 1.96) and 7.75 g decrease (95% CI -15.94, 0.44) in average birthweight among infants of women in the 2^nd^ tertile of the ½- and 2-mile metrics, respectively. Infants born to women classified in the 1^st^ and 3^rd^ tertiles of the 10-mile metric had birthweights, on average, 7.36 g (95% CI -14.79, 0.08) and 6.56 g (95% CI -13.68, 0.56) less than infants of women in the referent group.

**Table 4:** Crude and adjusted^1^ associations between UGD-activity and birthweight (grams), among 143,237 women with full-term births in the 24-county Barnett Shale area, Texas, Nov. 30, 2010—Nov. 29, 2012.

IDW Sum of UGD Well Activity	No.	Median (IQR) Wells	Crude β (95% CI)	Adjusted β (95% CI)
**½ Mile Buffer**				
0 Wells ≤10 mi	34,699		Reference	
1^st^ Tertile	7,479	1 (1–2)	29.63 (18.22, 41.04)	0.12 (-11.80, 12.04)
2^nd^ Tertile	7,693	4 (3–5)	17.30 (6.02, 28.58)	-8.20 (-18.36, 1.96)
3^rd^ Tertile	7,488	7 (5–10)	28.90 (17.50, 40.31)	-0.83 (-12.24, 10.58)
**2 Mile Buffer**				
0 Wells ≤10 mi	34,699		Reference	
1^st^ Tertile	21,373	7 (3–13)	30.11 (22.37, 37.86)	-4.39 (-12.33, 3.56)
2^nd^ Tertile	21,751	32 (23–42)	18.92 (11.21, 26.63)	-7.75 (-15.94, 0.44)
3^rd^ Tertile	21,303	54 (39–75)	24.17 (16.42, 31.93)	-6.68 (-14.38, 1.02)
**10 Mile Buffer**				
0 Wells ≤10 mi	34,699		Reference	
1^st^ Tertile	36,028	9 (1–66)	11.85 (5.15, 18.56)	-7.36 (-14.79, 0.08)
2^nd^ Tertile	36,845	418 (267–751)	25.65 (18.98, 32.31)	-2.58 (-9.75, 4.59)
3^rd^ Tertile	35,665	1191 (923–1492)	19.82 (13.10, 26.54)	-6.56 (-13.68, 0.56)

UGD: unconventional gas development; IDW: inverse distance weighted; IQR: interquartile range; CI: confidence interval; mi: miles.

^1^Model adjusted for maternal age at delivery, pre-pregnancy BMI, race/ethnicity, education, parity, smoking, adequacy of prenatal care utilization, previous poor pregnancy outcome, and infant sex.

Neither the additional adjustment for residential proximity to nearest major roadway nor season of conception resulted in meaningful changes to effect estimates (S1 and S2 Tables).

## Discussion

We found evidence of a moderate positive association between maternal residential proximity to UGD-activity and increased odds of preterm birth and a suggestive association with fetal death. Not surprisingly, we found that the characterization of UGD was dependent upon the distance within which activity was defined.

In contrast to our findings, a Colorado (CO) based study [19] reported an inverse association between UGD and preterm birth (3^rd^ tertile OR 0.91, 95% CI 0.85, 0.98) as well as a positive association with birthweight (3^rd^ tertile b 22, 95% CI 15, 29). The authors of that study defined UGD-activity based on active wells ≤10 miles of the maternal residence at any time during the child’s birth year. The authors also restricted the analysis to women in rural areas, given concerns of confounding by other sources of air pollution. A similar restriction was not feasible in the present study because the majority of UGD in the Barnett Shale occurs in urban/suburban areas. However, we did not observe meaningful changes in effect estimates when adjusting for proximity to the nearest major roadway, a marker for traffic-related air pollution.

Two studies of UGD and birth outcomes in Pennsylvania (PA) have conflicting results. Stacy et al. [20] employed an activity metric similar to that in the CO study [19]: all wells ≤10 miles of the maternal residence during the child’s birth year were included. In the second PA study, Casey et al. [21] included all wells in the state, regardless of their distance from the woman’s residence, but only considered UGD wells active during a woman’s pregnancy. Stacy et al. [20] found increased odds of SGA among women classified in the highest versus lowest UGD-activity quartile (OR 1.34; 95% CI 1.10, 1.63), but no association with preterm birth. In contrast, Casey et al. [21] reported increased odds of preterm birth among women in the 2^nd^ (OR 1.3, 95% CI 1.0, 1.8), 3^rd^ (OR 1.6, 95% CI 1.1, 2.4), and 4^th^ (OR 1.9, 95% CI 1.2, 2.9) UGD-activity quartiles, but no association with SGA. Stacy et al. [20] also found a 21.8 g decrease (p = 0.02) in birthweight among infants born to women in the highest versus lowest UGD-activity quartile. Though Stacy et al. [20] included gestational age in their birthweight models, we chose not to adjust for gestational age given its potential to act as a collider and thus, bias estimates of effect [33].

Given key differences in drilling characteristics (including density and distribution of wells in urban/rural areas) as well as population characteristics, it is difficult to directly compare our results with previous studies’ findings. For example, there were 509 total active wells in the Stacy et al. [20] study and the most highly ‘exposed’ women had ≥ six wells ≤10 miles of her home. Casey et al. [21] noted the greatest density of UGD near women’s homes was 122 wells ≤20 km. The maximum number of wells near women’s homes was not reported by McKenzie et al. [19]. Our study included >13,000 UGD wells and the median number of wells ≤10 miles of the residence of the most highly ‘exposed’ women was 1,188. Additionally, the racial/ethnic makeup of women in this study is quite different from previous studies, which have included primarily (73–97%) non-Hispanic white women [19–21]. Women in our study represent a more diverse population: 39.7% Hispanic, 16.1% Black, and 37.4% non-Hispanic white. Lastly, women in our study are from primarily urban/suburban areas compared with more rural populations in previous studies.

Both air and water contamination have been linked with UGD-activity, including UGD in the Barnett Shale. For example, air pollution models indicate urban drilling is a significant contributor to ambient ozone in the Barnett Shale [34], which may point to increased air toxics concentrations, given secondary formation of ozone through reactions between nitrogen oxides and VOCs [35]. Investigators have also demonstrated pollutant migration and groundwater contamination related to natural gas production in the Barnett Shale area [6]. In addition to chemical contamination, communities near UGD may be burdened by non-chemical stressors (see directed acyclic graph developed by Casey et al. [36]), which could affect health outcomes through altered allostatic load [37, 38]. In some areas, UGD occurs 24-hours a day and involves generator noise, increased truck traffic, noxious odors, and light pollution [15]. The temporarily increased workforce can lead to transient population growth with accompanying demands for goods and services [39]. UGD’s presence may also contribute to conflict and distrust, as well as division, within communities [40]. Such “boomtown” psychosocial effects have been indicated in UGD risk assessments as potential drivers of adverse health outcomes [11, 15, 31].

Endocrine disruption has been suggested as a possible mechanism through which UGD-related contaminants may increase risk of adverse perinatal outcomes including stillbirth, preterm birth, and decreased birthweight [9, 41, 42]. Some air pollutants may also affect preterm birth via oxidative stress, endothelial dysfunction, or inflammation [43, 44]. The maternal stress response (resulting from either chemical or non-chemical stressors) may also result in ‘dysregulated parturition’ and an altered ‘pregnancy clock’, ultimately leading to preterm delivery [45, 46]. The mechanism through which exposure to UGD-related contaminants may result in fetal death is less clear. It has been posited that some air pollutants may be directly transported across the placenta resulting in hypoxia or immune-mediated injury of the fetus [47]. Reduced oxygen-carrying capacity of maternal hemoglobin and alterations in transplacental function have also been suggested [48]. Fetuses may be spontaneously terminated among women in particularly stressful circumstances [49, 50], providing an additional mechanism through which fetal death may be impacted among women near UGD who experience chemical and non-chemical stressors.

One of the strengths of this study was the large sample size, even when considering UGD-activity relatively close to women’s homes. Though it is a rare outcome, our large sample size allowed us to explore the association between UGD-activity and fetal death, which other studies have not considered. It is possible that women with preterm births or fetal deaths may be assigned lower values for UGD-activity than women with term births simply due to their shorter gestational periods. However, we anticipate that the result of any such bias would be toward the null. Because spatial autocorrelation can lead to biased estimates when using spatially derived data [51], we also assessed potential impact of clustering of women within census-tracts using GEEs with an exchangeable error structure. Impact of maternal residential mobility may also result in bias. However, though Canfield et al. [52] report that approximately 32% of women in Texas change residence between conception and delivery, Lupo et al. [53] found, among women who do move, assignment of area-level exposure was not largely impacted by the use of residence at conception versus residence at delivery.

Though the exposure metric utilized in this study was non-specific, our goal was not to examine risk related to any specific chemical. Rather, it was to examine effects of living near increased UGD-activity, which encompasses potential exposure to a multitude of chemical and non-chemical stressors, through a variety of pathways. Our use of proximity to UGD-activity assumes that women who reside near wells are more likely to be exposed (or to be exposed at higher levels) to these stressors than women living farther away. Still, there is some uncertainty regarding the ideal distance within which to capture UGD-activity.

Prior studies evaluating maternal residential proximity to UGD and birth outcomes focused on UGD ≤10 miles of the maternal residence. However, we posit it may be more plausible for UGD-activity to affect perinatal outcomes (via increased chemical and non-chemical stressors) at a much smaller distance. Many air toxics, such as benzene, are highly volatile in the atmosphere and undergo degradation relatively quickly [54]–thus emissions of such pollutants are more likely to influence exposure of populations living near to, rather than far from, the source. McKenzie et al. [31] conducted an air pollution-focused risk assessment in Garfield County, CO and noted that residents ≤½-mile of a well pad had greater non-cancer health hazards than residents living further away. Results from a study near Dallas-Fort Worth indicated elevated modelled air toxics concentrations (e.g., acrolein, formaldehyde) near fence lines of wells (~600 feet), suggesting potential increased air pollution relatively near wells [12, 55]. To our knowledge, no published studies have evaluated personal exposure to UGD-related air pollution among individuals living varying distances from well sites. Nonetheless, a mechanism through which UGD-related chemical and non-chemical stressors increases risk of adverse health outcomes seems to have greater plausibility at more proximal distances. Thus, comprehensive exposure assessment studies are needed to inform relevant distances within which to best capture UGD-activity as it potentially relates to adverse health outcomes.

We found evidence of an association between maternal residential proximity to UGD-activity and preterm birth and limited evidence of an association with fetal death among a diverse population of women living near the Barnett Shale. Though there may be differences in air pollution emissions during completion and production phases of UGD drilling [10, 56], we defined UGD-activity metrics without regard to drilling phase. Thus, we are presently working toward estimation of phase-specific UGD-activity metrics to inform potential differences in perinatal health risks related to drilling phase. Additionally, we are pursuing methods to improve the assignment of exposure given potential differences in time-at-risk between preterm births and fetal deaths compared with term births. Nonetheless, the lack of detailed exposure assessment data remains a critical gap in understanding potential health risks associated with UGD-activity. Exposure assessment studies would serve to quantify chemical and non-chemical stressors to which residents living near UGD are exposed, validate UGD-activity metrics like the one used in this and previous studies [19–21], and inform the most relevant distances within which to characterize chemical and non-chemical stressors. Future priority should be placed on obtaining such information to better characterize risk and inform epidemiologic studies.

## Supporting information

S1 Table Sensitivity analyses for preterm birth, SGA, and fetal death. (DOCX)

S2 Table Sensitivity analyses for birthweight. (DOCX)
