# Supplementary material for: Maternal residential proximity to unconventional gas development and perinatal outcomes among a diverse urban population in Texas
Source: PLoS One. 2017 Jul 21;12(7):e0180966. doi: 10.1371/journal.pone.0180966 (PMC5522007; doi:10.1371/journal.pone.0180966)
Supplement: S1 Table — (DOCX) [file pone.0180966.s001.docx]

| **Table S1.** Adjusted^1^ odds ratios for the association between unconventional gas drilling activity during gestation and adverse birth outcomes, among 156,697 women with a birth or fetal death in the Barnett Shale, Nov. 30, 2010 - Nov. 29, 2012. | | | | |
| --- | --- | --- | --- | --- |
|  |  |  |  |  |
|  |  |  |  |  |
|  |  |  |  |  |
| IDW Sum of UGD Well Activity | No. | Cases | Adjusted + Major Roadway OR (95% CI) | Adjusted + Season of Conception  OR (95% CI) |
|  |  |  |  |  |
|  |  |  |  |  |
| ½ *Mile Buffer* | | | | |
| **Preterm Birth^2^** |  |  |  |  |
| 0 Wells ≤10 mi | 37,885 | 3,183 | Reference | |
| 1^st^ Tertile | 8,161 | 682 | 1.18 (1.08, 1.30) | 1.18 (1.08, 1.29) |
| 2^nd^ Tertile | 8,412 | 717 | 1.21 (1.10, 1.33) | 1.20 (1.09, 1.33) |
| 3^rd^ Tertile | 8,144 | 654 | 1.14 (1.03, 1.25) | 1.14 (1.03, 1.25) |
| **SGA^3^** |  |  |  |  |
| 0 Wells ≤10 mi | 37,882 | 5,361 | Reference | |
| 1^st^ Tertile | 8,161 | 964 | 0.91 (0.84, 0.98) | 0.91 (0.84, 0.98) |
| 2^nd^ Tertile | 8,409 | 1,063 | 0.96 (0.90, 1.03) | 0.97 (0.90, 1.04) |
| 3^rd^ Tertile | 8,142 | 1,013 | 0.96 (0.90, 1.04) | 0.97 (0.90, 1.04) |
| **Fetal Death^4^** |  |  |  |  |
| 0 Wells ≤10 mi | 38,029 | 147 | Reference | |
| 1^st^ Tertile | 8,188 | 27 | 1.07 (0.69, 1.65) | 1.06 (0.69, 1.64) |
| 2^nd^ Tertile | 8,438 | 29 | 1.11 (0.72, 1.71) | 1.09 (0.71, 1.69) |
| 3^rd^ Tertile | 8,174 | 32 | 1.27 (0.82, 1.97) | 1.26 (0.82, 1.96) |
| *2 Mile Buffer* | | | | |
| **Preterm Birth^2^** |  |  |  |  |
| 0 Wells ≤10 mi | 37,885 | 3,183 | Reference | |
| 1^st^ Tertile | 23,231 | 1,856 | 1.11 (1.04, 1.19) | 1.11 (1.04, 1.19) |
| 2^nd^ Tertile | 23,758 | 2,006 | 1.16 (1.09, 1.24) | 1.16 (1.09, 1.24) |
| 3^rd^ Tertile | 23,227 | 1,921 | 1.14 (1.07, 1.22) | 1.14 (1.07, 1.22) |
| **SGA^3^** |  |  |  |  |
| 0 Wells ≤10 mi | 37,882 | 5,361 | Reference | |
| 1^st^ Tertile | 23,227 | 2,785 | 0.94 (0.90, 1.00) | 0.95 (0.90, 1.00) |
| 2^nd^ Tertile | 23,757 | 2,984 | 0.96 (0.91, 1.01) | 0.96 (0.91, 1.01) |
| 3^rd^ Tertile | 23,223 | 2,847 | 0.95 (0.90, 1.00) | 0.95 (0.90, 1.00) |
| **Fetal Death^4^** |  |  |  |  |
| 0 Wells ≤10 mi | 38,029 | 147 | Reference | |
| 1^st^ Tertile | 23,301 | 74 | 1.14 (0.83, 1.56) | 1.14 (0.83, 1.56) |
| 2^nd^ Tertile | 23,860 | 103 | 1.56 (1.16, 2.11) | 1.56 (1.15, 2.10) |
| 3^rd^ Tertile | 23,300 | 77 | 1.16 (0.86, 1.58) | 1.15 (0.85, 1.57) |
| *10 Mile Buffer* | | | | |
| **Preterm Birth^2^** |  |  |  |  |
| 0 Wells ≤10 mi | 37,885 | 3,183 | Reference | |
| 1^st^ Tertile | 39,169 | 3,140 | 1.02 (0.96, 1.08) | 1.02 (0.96, 1.08) |
| 2^nd^ Tertile | 40,143 | 3,296 | 1.13 (1.06, 1.20) | 1.13 (1.06, 1.20) |
| 3^rd^ Tertile | 38,922 | 3,253 | 1.15 (1.08, 1.21) | 1.15 (1.09, 1.22) |
| **SGA^3^** |  |  |  |  |
| 0 Wells ≤10 mi | 37,882 | 5,361 | Reference | |
| 1^st^ Tertile | 39,168 | 5,233 | 1.02 (0.99, 1.08) | 1.03 (0.98, 1.08) |
| 2^nd^ Tertile | 40,139 | 4,924 | 0.95 (0.91, 0.99) | 0.95 (0.91, 1.00) |
| 3^rd^ Tertile | 38,917 | 4,877 | 0.96 (0.92, 1.00) | 0.96 (0.92, 1.00) |
| **Fetal Death^4^** |  |  |  |  |
| 0 Wells ≤10 mi | 38,029 | 147 | Reference | |
| 1^st^ Tertile | 39,325 | 157 | 1.26 (0.99, 1.60) | 1.26 (0.99, 1.60) |
| 2^nd^ Tertile | 40,277 | 138 | 1.22 (0.95, 1.57) | 1.22 (0.94, 1.57) |
| 3^rd^ Tertile | 39,066 | 149 | 1.34 (1.05, 1.72) | 1.34 (1.04, 1.72) |
| UGD: unconventional gas development; IDW: inverse distance weighted; IQR: interquartile range; OR: odds ratio; CI: confidence interval; mi: miles. | | | | |
|  |  |  |  |  |
| ^1^All models adjusted for maternal age at delivery, pre-pregnancy BMI, race/ethnicity, education, smoking, adequacy of prenatal care utilization, and infant sex. | | | | |
|  |  |  |  |  |
|  |  |  |  |  |
| ^2^Preterm Birth models additionally adjusted for previous poor pregnancy outcome; n = 156,119: fetal deaths excluded from analyses. | | | | |
|  |  |  |  |  |
| ^3^SGA models additionally adjusted for parity; n=156,106: fetal deaths excluded from analyses. | | | | |
| ^4^Fetal Death models additionally adjusted for parity & previous poor pregnancy outcome. | | | | |
