# Supplementary material for: Maternal residential proximity to unconventional gas development and perinatal outcomes among a diverse urban population in Texas
Source: PLoS One. 2017 Jul 21;12(7):e0180966. doi: 10.1371/journal.pone.0180966 (PMC5522007; doi:10.1371/journal.pone.0180966)
Supplement: S2 Table — (DOCX) [file pone.0180966.s002.docx]

| **Table S2.** Adjusted associations between UGD-activity and birthweight (g), among 143,237 women with full-term births in the 24-county Barnett Shale area, Texas, Nov. 30, 2010 - Nov. 29, 2012. | | | | | | | |
| --- | --- | --- | --- | --- | --- | --- | --- |
|  |  |  |  |  |  |  |  |
|  |  |  |  |  |  |  |  |
| IDW Sum of UGD Well Activity | No. | Adjusted^1^ + Major Roadway β (95% CI) | | | Adjusted^1^ + Season of Conception  β (95% CI) | | |
|  |  |  |  |  |  |  |  |
| **½ Mile Buffer** |  |  |  |  |  |  |  |
| 0 Wells ≤10 mi | 34,699 | Reference | | | | | |
| 1^st^ Tertile | 7,479 | 0.28 (-11.69, 12.25) | | | 0.16 (-11.76, 12.07) | | |
| 2^nd^ Tertile | 7,693 | -7.99 (-18.15, 2.18) | | | -8.10 (-18.26, 2.07) | | |
| 3^rd^ Tertile | 7,488 | -0.68 (-12.12, 10.76) | | | -0.72 (-12.14, 10.70) | | |
| **2 Mile Buffer** |  |  |  |  |  |  |  |
| 0 Wells ≤10 mi | 34,699 | Reference | | | | | |
| 1^st^ Tertile | 21,373 | -4.22 (-12.20, 3.75) | | | -4.38 (-12.32, 3.56) | | |
| 2^nd^ Tertile | 21,751 | -7.55 (-15.76, 0.67) | | | -7.75 (-15.94, 0.44) | | |
| 3^rd^ Tertile | 21,303 | -6.56 (-14.28, 1.16) | | | -6.69 (-14.40, 1.02) | | |
| **10 Mile Buffer** |  |  |  |  |  |  |  |
| 0 Wells ≤10 mi | 34,699 | Reference | | | | | |
| 1^st^ Tertile | 36,028 | -7.36 (-14.79, 0.07) | | | -7.36 (-14.79, 0.08) | | |
| 2^nd^ Tertile | 36,845 | -2.41 (-9.59, 4.78) | | | -2.58 (-9.75, 4.58) | | |
| 3^rd^ Tertile | 35,665 | -6.39 (-13.52, 0.74) | | | -6.56 (-13.68, 0.56) | | |
| UGD: unconventional gas development; IDW: inverse distance weighted; No.: number; CI: confidence interval; mi: miles. | | | | | | | |
|  |  |  |  |  |  |  |  |
| ^1^All models adjusted for maternal age at delivery, pre-pregnancy BMI, race/ethnicity, education, smoking, adequacy of prenatal care utilization, parity, previous poor pregnancy outcome, and infant sex. | | | | | | | |
|  |  |  |  |  |  |  |  |
|  | | | | | | | |
|  |  |  |  |  |  |  |  |
